# The Unfolded Protein Response: A Double-Edged Sword for Brain Health

**DOI:** 10.3390/antiox12081648

**Published:** 2023-08-21

**Authors:** Magdalena Gebert, Jakub Sławski, Leszek Kalinowski, James F. Collawn, Rafal Bartoszewski

**Affiliations:** 1 Department of Medical Laboratory Diagnostics—Fahrenheit Biobank BBMRI.pl, Medical University of Gdansk, 80-134 Gdansk, Poland; 2 Department of Biophysics, Faculty of Biotechnology, University of Wroclaw, F. Joliot-Curie 14a Street, 50-383 Wroclaw, Poland; 3 BioTechMed Centre, Department of Mechanics of Materials and Structures, Gdansk University of Technology, 11/12 Narutowicza Street, 80-233 Gdansk, Poland; 4 Department of Cell, Developmental, and Integrative Biology, University of Alabama at Birmingham, Birmingham, AL 35233, USA

**Keywords:** endoplasmic reticulum stress, mitochondria unfolded protein response, oxidative stress, neurodegeneration, proteostasis, calcium, brain, nitrosative stress, oxygen homeostasis

## Abstract

Efficient brain function requires as much as 20% of the total oxygen intake to support normal neuronal cell function. This level of oxygen usage, however, leads to the generation of free radicals, and thus can lead to oxidative stress and potentially to age-related cognitive decay and even neurodegenerative diseases. The regulation of this system requires a complex monitoring network to maintain proper oxygen homeostasis. Furthermore, the high content of mitochondria in the brain has elevated glucose demands, and thus requires a normal redox balance. Maintaining this is mediated by adaptive stress response pathways that permit cells to survive oxidative stress and to minimize cellular damage. These stress pathways rely on the proper function of the endoplasmic reticulum (ER) and the activation of the unfolded protein response (UPR), a cellular pathway responsible for normal ER function and cell survival. Interestingly, the UPR has two opposing signaling pathways, one that promotes cell survival and one that induces apoptosis. In this narrative review, we discuss the opposing roles of the UPR signaling pathways and how a better understanding of these stress pathways could potentially allow for the development of effective strategies to prevent age-related cognitive decay as well as treat neurodegenerative diseases.

## 1. Introduction

Proper oxygen (O_2_) homeostasis is essential for human survival, and the human brain consumes about 20% of the total oxygen to support neurons and glia [1,2,3,4]. Unmet brain oxygen needs during ischemic stroke limit ATP synthesis [5,6]. Oxygen consumption results in the generation of free radicals and non-radicals including superoxide (O_2_^.-^) and hydroxyl anions (^.^OH), and hydrogen peroxide (H_2_O_2_) [7,8,9,10]. Although this is an unavoidable consequence of oxygen-dependent brain activity, if not controlled properly, it leads to oxidative stress and neurodegeneration [11,12,13,14,15,16,17,18,19]. Thus, maintaining proper oxygen homeostasis in brain tissues requires a balanced level of O_2_-derived free radicals and non-radicals [1]. In this review, we discuss how the unfolded protein response (UPR) regulates oxygen homeostasis in the endoplasmic reticulum (ER) and mitochondria to support neuronal cell viability, but also how these stress pathways can promote cognitive decline and potentially neuronal diseases.

Given that maintaining the redox balance is necessary for cell survival, it is surprising that the brain is so susceptible to oxidative stress and oxidative damage [1]. This vulnerability to brain oxygen damage is believed to be a compromise between brain function and the biochemical organization that is required for survival [20]. This organization includes a high content of mitochondria, an increased glucose demand, and a high influx of neuronal Ca^2+^. Furthermore, there is increased microglia activity, as well as increased neuronal nitric oxide synthase (nNOS) and nicotinamide adenine dinucleotide phosphate (NAPDH) oxidase (NOX) signaling, along with the presence of autoxidizable neurotransmitters. This metabolism machinery generates hydrogen peroxide, high concentrations of peroxidable lipids, elevated levels of cytochrome P_450_, and the enrichment of brain tissues in redox-active transition metals such as Fe^2+^ and Cu^+^ [1,11,12,13,14,15,16,17,18,19,21,22]. All of this leads to potential stress that needs to be properly and safely regulated.

In this complex system, brain cells have to efficiently modulate their signaling pathways to maintain their redox balance and utilize universal adaptive stress responses in order to survive periods of elevated oxidation levels and minimize cellular damage. These stress pathways depend on the proper function of the endoplasmic reticulum (ER) and activation of the unfolded protein response (UPR), a set of complex molecular pathways that regulate proper ER function required for cell survival, or in the case of unmitigated cell stress, lead to cell death. In this review, we discuss the Janus faces of this complex signaling pathway in the context of managing the “oxidant burden” of the brain [23,24].

## 2. Role of the ER in Maintaining Neuron Cell Homeostasis

### 2.1. Calcium Regulation and Signaling

Connecting synaptic activity with the biochemical signals of neurons occurs through utilizing calcium ions (Ca^2+^) as the main second messenger to regulate activity-dependent signaling [25,26]. Brain calcium fluxes lead to high ATP demands that restore the ion levels after calcium influx through the plasma membrane receptor. When impaired, intracellular calcium homeostasis leads to increased generation of mitochondrial reactive oxygen species (ROS) [27]. The ER, the main cellular calcium storage compartment, remains a critical system responsible for the calcium balance in neurons [28]. ER calcium release in response to small increases in its cytosolic levels is termed calcium-induced calcium release (CICR), whereas the reduction in calcium concentration in ER lumen is referred to as storage-operated calcium entry (SOCE) [28]. Both of these mechanisms amplify cytosolic calcium levels and allow the ER, at least in theory, to generate calcium transients independently of any plasma membrane depolarization [29]. Furthermore, ER calcium release and uptake in neurons relies on the membrane potential and contributes to its modulation by accelerating increases and decreases in the calcium cytosolic levels.

The excessive influx of calcium into neurons mainly occurs through the activation of N-methyl-D-aspartate (NMDA) receptors by glutamate, and results in CICR [28]. Although the influx of calcium through NMDA receptors is the underlying basis of neurodegeneration caused by excitotoxicity, calcium stores within the endoplasmic reticulum (ER) can also be released through ryanodine receptors (RyR) and inositol 1,4,5-trisphosphate receptors (IP_3_R) under these conditions, and this can amplify the pathological calcium signals [28,29]. As a consequence, the activation of the mitochondrial calcium buffering system can occur and lead to rapid mitochondrial damage due to increased permeability of the transition pore (mPTP) [28,30,31]. Furthermore, the increase in intracellular calcium concentration is accompanied by O_2_^-^ release and the generation of OH^.^ in the Fenton reaction, which is catalyzed by superoxide dismutase (SOD) [32,33].

ER calcium release in the region of mitochondria-associated membranes (MAMs) [34,35] has been shown to support the ATP demand-related mitochondrial uptake of calcium [36,37]. Mitochondrial calcium uptake leads to increases in the activity of the Krebs cycle enzymes [36,37,38,39]. Despite multiple pathways that allow mitochondrial calcium release that include both ion exchangers and the transient opening of the mitochondrial permeability transition pore (mPTP) [30,31], mitochondria remain prone to calcium overload. This unfortunately leads to reduced ATP synthesis, increased ROS formation [40,41], and eventually cell death [42]. This highlights the importance of the cooperation between mitochondria and ER in regulating intracellular calcium levels and neuronal cell viability.

### 2.2. The ER and Proteostasis

The spatial organization of the brain dependence on this complex neuronal structure is maintained by the continuous protein profile-related remodeling of synapses [43,44,45]. Their proper function relies on the biogenesis of plasma membranes that are enriched with specific proteins, including cell adhesion molecules, ion channels, receptors, and transporters [46]. The ER is a central compartment for the secretory protein pathway, which is important for membrane protein maturation and lipid biosynthesis, and this pathway remains critical both during and after brain development [47,48]. Proper ER functions are crucial for both synapse formation and plasticity as well for cognitive functions [47,48,49,50,51].

The ER also contains enzymes and chaperones that assist in various protein folding scenarios and mediates their posttranslational maturation [52]. This protein maturation machinery includes chaperone immunoglobulin binding protein (BiP; also known as *HSPA5* or Grp78) [53], different oxidoreductases of the protein disulfide isomerase (PDI) family [54], and the peptidyl prolyl cis-trans isomerases (PPIs) [55]. Protein quality control of the ER-maturating glycosylated proteins is ensured by the calnexin–calreticulin system [56], whereas terminally misfolded peptides are exported from the ER and degraded either by the proteasome (ER-associated degradation (ERAD)) or the lysosome (ER-to-lysosome-associated degradation (ERLAD)) [57,58]. Random oxidation of mRNA is one of the consequences of the brain oxygen burden [59], and this can increase translational errors [60], reduce the successful protein folding in ER [61,62,63], and provide challenges for the ER-associated degradation system. Furthermore, impaired efficiency of ER-related protein maturation can result in deregulation of brain redox homeostasis and lead to oxidative damage. Oxidative stress can also impair ER proteostasis and ER-associated degradation, leading to accumulation and aggregation of misfolded proteins, as is observed during neurodegeneration [64,65].

### 2.3. The ER Lipid Biosynthesis

ER-localized enzymes are also responsible for the synthesis of the majority of cellular lipids that are another key component of the brain. These membrane lipids allow the brain cells to grow, proliferate, differentiate, and modulate neurons and glia cell function, including neurotransmission [66,67,68]. Interestingly, the brain is enriched in long-chain polyunsaturated fatty acids that are sensitive to oxidation, but neurons do not store energy in the form of glycogen or lipid droplets. Therefore, fatty acid oxidation primarily occurs in astrocytes that transfer the related metabolites to neurons [69]. Furthermore, stressed neurons release peroxidated fatty acids to be endocytosed and stored in lipid droplets by neighboring astrocytes that utilize this storage to support the stimulated neuron energy requirements [70]. This lipid crosstalk between the neurons and astrocytes ensures proper brain function, while minimizing the risk of oxidative stress [69]. This cooperation between the neurons and astrocytes prevents a buildup of peroxidated fatty acids in neurons during periods of prolonged stimulation [70].

Cholesterol, on the other hand, is enriched in synaptic membranes and serves as a regulator of neurotransmissions. It is synthetized de novo in both neurons and astrocytes [71,72]. The cholesterol synthesis pathway is dependent upon the ER-associated sterol regulatory element-binding protein (SREBP) system that is activated by low cholesterol levels in ER membranes and is very sensitive to the alterations in ER homeostasis [73,74].

**Figure 1:**
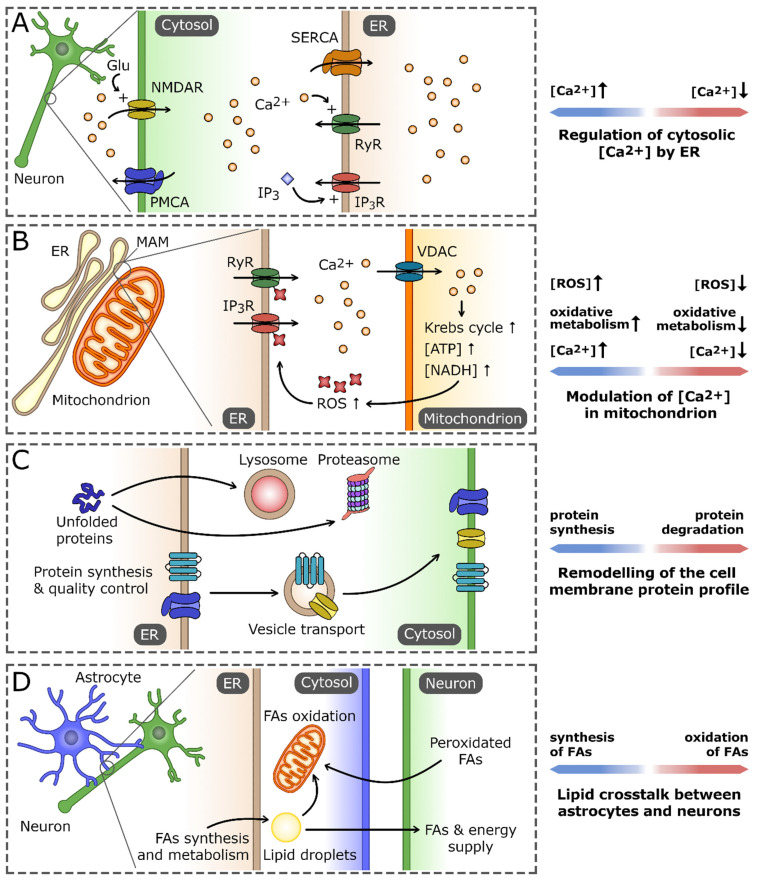
The role of the endoplasmic reticulum (ER) in maintaining neuron cell homeostasis. (**A**) As the main Ca^2+^ reservoir, the ER is crucial for the regulation of cytosolic Ca^2+^ concentration using pumps and channels localized in ER membrane. Those include sarco/endoplasmic reticulum Ca^2+^ ATPase (SERCA), Ca^2+^-activated ryanodine receptors (RyRs), and inositol-1,4,5-trisphosphate (IP_3_)-gated IP_3_ receptors (IP_3_Rs). They cooperate with the cell membrane Ca^2+^ transporters that regulate the influx of extracellular Ca^2+^, exemplified by plasma membrane Ca^2+^ ATPase (PMCA) and N-methyl-D-aspartate receptor (NMDAR). (**B**) Ca^2+^ homeostasis processes in the ER and mitochondrion are tightly interconnected, primarily by virtue of the regions of mitochondria-associated membranes (MAMs). An increase in Ca^2+^ concentration in MAM promotes its influx into the mitochondrion, mainly through voltage-dependent anion channel (VDAC). High Ca^2+^ concentration stimulates the activity of the oxidative processes in the mitochondrion, leading to the increased production of reactive oxygen species (ROS). In turn, ROS-dependent modifications of ER Ca^2+^ channels increase their permeability for Ca^2+^ and the efflux of Ca^2+^ from ER, which closes the positive-feedback loop. (**C**) The ER is a central cell compartment where the synthesis and quality control of secretory and membrane proteins takes place. The properly folded proteins are directed through secretory pathway to the cell membrane, whereas irreversibly unfolded/misfolded proteins are exported and eventually degraded either in lysosomes or proteasomes. (**D**) ER-based lipid crosstalk between neurons and astrocytes. Fatty acids (FAs) and the products of their oxidation synthesized in astrocytes are delivered to neurons to support their demand for energy and membrane building components. In turn, nonfunctional peroxidated FAs released by neurons are endocytosed by astrocytes and stored in lipid droplets or catabolized by the mitochondrial FA oxidation pathway.

In summary, ER homeostasis (as presented in Figure 1) remains one of the key factors for brain development and function, including the redox balance. ER homeostasis is stabilized by the presence of the UPR. The UPR promotes cellular survival by reducing ER damage during stress, or alternatively promotes cell death during prolonged or unmitigated stress [75]. This negative scenario is a common characteristic of neurodegenerative diseases caused by aggregates of mutant proteins or through loss of function of genes responsible for proteostasis [75,76,77,78]. Thus, the ability of UPR to determine cell fate is a crucial element of brain aging and potential neurodegeneration.

## 3. The Unfolded Protein Response Pathway

**Figure 2:**
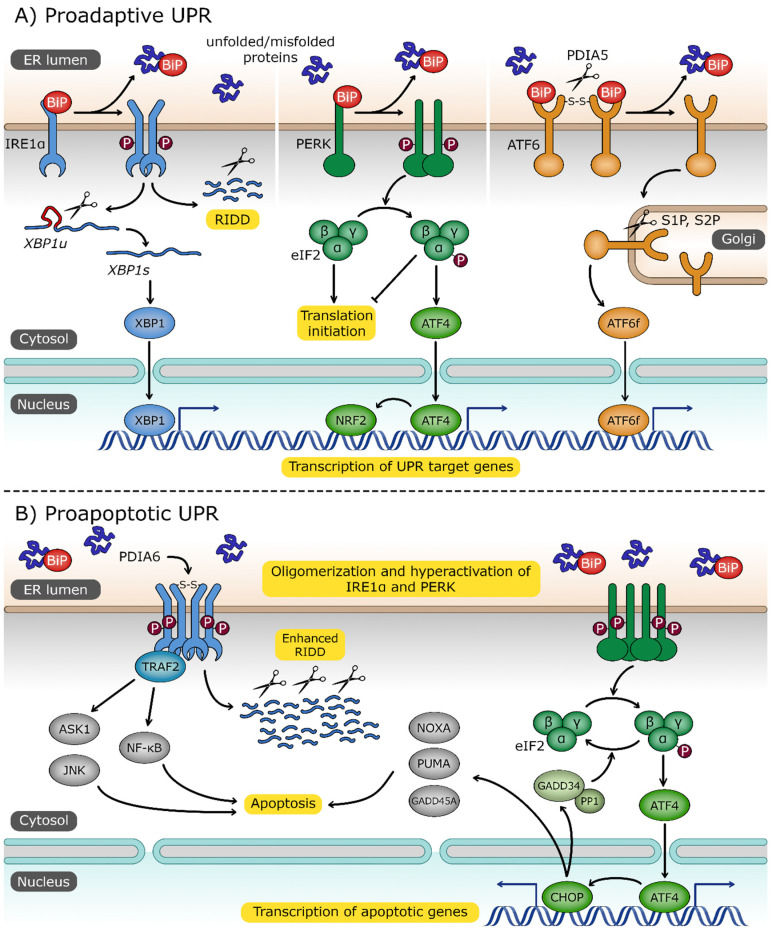
The unfolded protein response (UPR) pathway. (**A**) Three UPR sensors—inositol-requiring protein 1α (IRE1α), protein kinase RNA (PKR)-like endoplasmic reticulum kinase (PERK) and activating transcription factor 6 (ATF6)—are localized in endoplasmic reticulum (ER) membrane and share a common activation signal: the dissociation of binding immunoglobulin protein (BiP) chaperone in response to increased level of unfolded/misfolded proteins. Dimerization of IRE1α, followed by its trans-autophosphorylation, activates its RNase domain. The primary target of IRE1α is the unspliced X box-binding protein 1 (XBP1u) transcript. Spliced XBP1 mRNA (XBP1s) encodes transcription factor XBP1s, which activates UPR-associated genes. IRE1α also degrades certain mRNAs through the regulated IRE1-dependent decay (RIDD) process. Upon dimerization and trans-autophosphorylation, PERK phosphorylates eukaryotic translation initiator factor 2α (eIF2α) to attenuate general protein translation. Phosphorylated eIF2α promotes expression of activating transcription factor 4 (ATF4) and nuclear factor erythroid 2-related factor 2 (NRF2), which are involved in the response to ER and oxidative stress, respectively. ER stress triggers the cleavage of disulfide bonds, stabilizing ATF6 oligomers by protein disulfide isomerase family A member 5 (PDIA5), and this is followed by its transport to the Golgi apparatus where it is processed by site 1 and site 2 proteases (S1P, S2P). Cytosolic ATF6 fragment (ATF6f) is released and imported to the nucleus, where it plays the role of an active transcription factor. (**B**) Under extensive and persistent ER stress, the UPR switches from proadaptive to a proapoptotic character. Oligomerized IRE1α, stabilized by the disulfide bonds formed by protein disulfide isomerase family A member 6 (PDIA6), recruits tumor necrosis factor (TNF) receptor-associated factor 2 (TRAF2), which in turn activates the proapoptotic signal-regulating kinase 1/Janus N-terminal kinase (ASK1/JNK) and the nuclear factor kappa-light-chain-enhancer of activated B cells (NF-κB) signaling pathways. ATF4 promotes the expression of CCAAT/enhancer-binding protein homologous protein (CHOP) and transcription factor targeting apoptotic genes, including growth arrest and DNA damage-inducible 45 alpha (GADD45A), p53 upregulated modulator of apoptosis (PUMA), phorbol-12-myristate-13-acetate-induced protein 1 (NOXA), and growth arrest and DNA damage-inducible 34 (GADD34). GADD34 forms a complex with protein phosphatase 1 (PP1) to dephosphorylate eIF2α and reverse the inhibition of translation.

The proper ratio between folded and unfolded proteins in the ER is an essential component of ER homeostasis [79]. Nevertheless, numerous cellular and environmental and physiological insults, including gene mutations, prion transmission, virial infections and ROS, promote ER stress. This results in the extensive accumulation of misfolded or incompletely folded proteins in the lumen of this organelle [75,76,77,78,80,81,82,83,84,85,86,87]. This type of disturbance of proteostasis calls for reductions in the protein synthetic load and increases in the availability of ER chaperones such as BiP [88]. Consequently, the pool of BiP associated with the ER UPR transmembrane proteins is released into the ER lumen to facilitate folding while simultaneously activating the UPR proteins (Figure 2A). These UPR proteins include protein kinase RNA (PKR)-like ER kinase (PERK), inositol-requiring transmembrane kinase/endoribonuclease (IRE1α), and activating transcription factor 6 (ATF6)) [89]. After BiP release, both IRE1 and PERK self-associate and undergo trans-autophosphorylation to become functional [88,89,90,91], whereas ATF6 translocates to the Golgi, where it is subjected to intermembrane proteolysis by site 1 and 2 proteases, yielding the nuclear-targeted transcription factor ATF6f (p50) [92,93,94,95].

PERK phosphorylates an alpha subunit of the eukaryotic initiation factor 2 (eIF2α), yielding P-eIF2α [96,97]. This in turn reduces the global rates of protein synthesis by inhibiting the activity of its own guanine nucleotide exchange factor [98]. The PERK-mediated reduction in cellular protein synthesis, referred to as the integrated stress response (ISR), reduces the ER peptide influx and allows correction of the degradation of misfolded proteins [99,100,101,102]. Nevertheless, the ISR-related translational blockage does not apply to the translation of a limited number of specific genes, including the growth arrest and DNA damage-inducible protein (GADD34), proapoptotic CCAAT/enhancer binding homologous protein (CHOP), and activating transcription factor 4 (ATF4) [89,103,104,105,106]. ATF4 enhances expression of antiapoptotic factors as well as—along with nuclear factor erythroid 2–related factor 2 (NRF2)—modulates glutathione (GSH) synthesis and the response to oxidative stress [107,108]. If the ER stress is diminished, GADD34 dephosphorylates P-eIF2α and thus reverses the translational blockage when the stress response is resolved [109].

Upon trans-autophosphorylation, IRE1’s endoribonuclease (RNase) activity is initiated, which allows it to degrade a subset of mRNAs to reduce the ER load of newly translated proteins in a process called IRE1-dependent decay (RIDD) [106,110]. Secondly, IRE1 splices the mRNA transcript of the X-box binding protein 1 (XBP1) transcription factor into an mRNA that encodes a transcriptionally active isoform of this protein (XBP1s) [111].

Both ATF6f and the XBP1s mediate a wide transcriptional reprogramming of stressed ER cells. These transcription factors work both cooperatively and independently to reduce ER peptide influx, increase folding processes in ER, and improve misfolded protein removal [82,112,113,114]. Furthermore, both ATF6f and XBP1s stimulate ER lipid membrane biosynthesis and chaperone transcription to increase the volume and folding capacity of the ER. They also promote the expression of the genes responsible for ERAD, including synoviolin 1 (HRD1), which is XBP1-induced, and the suppressor/enhancer of lin-12-like (SEL1L), which is induced by both ATF6f and XBP1s [115,116,117] and N-glycosylation [82,98,118,119,120]. Notably, ATF6f and XBP1s transcriptional targets include prosurvival transcripts [111,114,118,121,122]. Although the ER requires increased production of membrane lipids in order to increase the ER volume during the UPR, this approach remains the most straightforward mechanism for the cell to resolve the stress and improve protein folding [123]. Despite the fact that all of the UPR branches stimulate lipid biogenesis [120,124,125,126], XBP1s remain the most critical for efficient increasing the ER volume [127,128,129].

The UPR can also realign its three signaling branches towards cell death programs (Figure 2B). The UPR-related cell death shifts the balance away from the proadaptive signals in cases where the cellular damage is too severe or the adaptative response fails [114,130,131]. Both PERK and ATF6f continuously stimulate expression of CHOP, whereas IRE1 leads to the activation of the Janus N-terminal kinase (JNK) [130,132,133,134]. The RIDD allows for the accumulation of proapoptotic factors by degrading their specific miRNAs that target these factors [135,136]. Furthermore, upon eventual hyperactivation of IRE1, in addition to RIDD, this RNAse forms a scaffold for the activation of proinflammatory and apoptotic ASK1-JNK and NF-kβ pathways [137,138]. IRE1-ASK1-JNK signaling leads to the inhibition of mitochondrial respiration and enhanced ROS production [139]. Interestingly, IRE1 activation can also prevent the proapoptotic activity of ATF6f [140].

The UPR cell death decision is also supported by changes in levels of other apoptotic factors such as growth arrest and DNA damage-inducible alpha (GADD45A), p53 upregulated modulator of apoptosis (PUMA), and phorbol-12-myristate-13-acetate-induced protein 1 (PMAIP1, also known as NOXA) [82,130,131,141,142,143,144]. Notably, PUMA and NOXA provide the link between UPR-induced cell death and mitochondrial apoptosis [145]. Since these two proteins contribute to the outer mitochondrial membrane permeabilization, their accumulation during ER stress can result in enhanced ROS efflux from mitochondria and accelerated oxidative stress [146]. Furthermore, if cells are exposed to strong and chronic ER insults, potent activation of PERK signals will result in a rapid decline in ATP levels accompanied by an intensive release of ER-stored calcium that leads to necroptosis [147,148,149,150,151,152]. Notably, necroptosis is also often associated with increased ROS levels [153,154,155,156]. It is also worth mentioning that both proadaptive and apoptotic aspects of the UPR are modulated at the posttranscriptional levels by the accompanying ER stress specific changes in noncoding RNAs, especially microRNAs [114,121,131,136,157,158,159,160,161,162,163,164,165].

## 4. The Mitochondrial UPR

Since mitochondria play a central role in terms of ROS-produced oxidative stress in brain, the impairment of ATP production and deregulation of mitochondrial function may also deregulate protein import and homeostasis in these organelles, and result in the induction of the mitochondrial UPR (UPRmt) [166,167,168,169]. In order to respond to such an insult, the mitochondrial UPR pathway has to adjust both mitochondria and nuclear encoded genes in order to increase the levels of ROS scavengers and mitochondrial chaperones and proteases. Chronic stress can lead to apoptosis [166,167,168,169,170].

It has been suggested that the mitochondrial UPR can serve as a protective mechanism against ATP depletion, mitochondrial protein misfolding or loss of mitochondrial inner membrane potential [168,171]. For example, the activation of UPRmt favors glycolysis [170,172], while at the same time it stimulates mitochondrial ROS removal [168]. The UPRmt has also been associated with a number of human diseases, including cancers, cardiac pathophysiology, neurodegeneration and Alzheimer’s disease [168,171,173,174,175].

While significant progress on deciphering the UPRmt mechanisms was achieved initially in *C. elegans*, it is only recently that the human UPRmt has become better characterized [176]. It has been shown, for example, that the UPRmt can result in the activation of the PERK axis of the UPR and thus increase levels of ATF4, ATF5 and CHOP as well as participate in ISR [166,167,168,169,177,178,179,180]. Mitochondrial disfunction has also been shown to lead to eIF2 phosphorylation, and this promotes the translation of ATF4, CHOP and activating transcription factor 5 (ATF5). These factors stimulate the transcription of the genes responsible for the recovery from mitochondrial insults including the mitochondrial chaperones [166,168,169,178,179,180]. ATF4 induces the transcription of the supercomplex assembly factor 1 (*SCAF1*) that supports OXPHOS metabolic reprograming [181]. Furthermore, ATF5 serves as sensor of mitochondrial homeostasis since its activity is inhibited when the protein import into healthy mitochondria is restored [182]. Since ATF5 contains both a mitochondrial translocation signal and a nuclear localization signal. During non-stress conditions, it is selectively imported into mitochondria for subsequent degradation by resident proteases [182].

Depending on the cause of mitochondrial dysfunction, different kinases can phosphorylate eIF2 [176]. Besides the ER stress and oxidative stress-related PERK kinase, eIF2 can be also phosphorylated by ribosome-associated general control nonderepressible 2 (GCN2) during stalled translation [183,184], whereas in the absence of heme or with the binding of the death ligand signal enhancer (DELE1), a mitochondrial protein that is exported to cytosol during stress, the eIF2 heme-regulated inhibitor (HRI) is activated [176,185,186]. Furthermore, eIF2 can also be phosphorylated by protein kinase R (PKR) activated by mitochondrial matrix-generated dsRNA [187]. Interestingly, the ISR-related translational blockage includes blocking the synthesis of the mitochondrial subunits of the channels responsible for protein import to attenuate mitochondrial stress [176,188,189]. Given the importance of mitochondrial homeostasis in the brain, understanding the crosstalk between the mitochondrial and the ER UPR pathways will require further study [190].

## 5. Oxidative Insults Can Cause ER Stress

**Figure 3:**
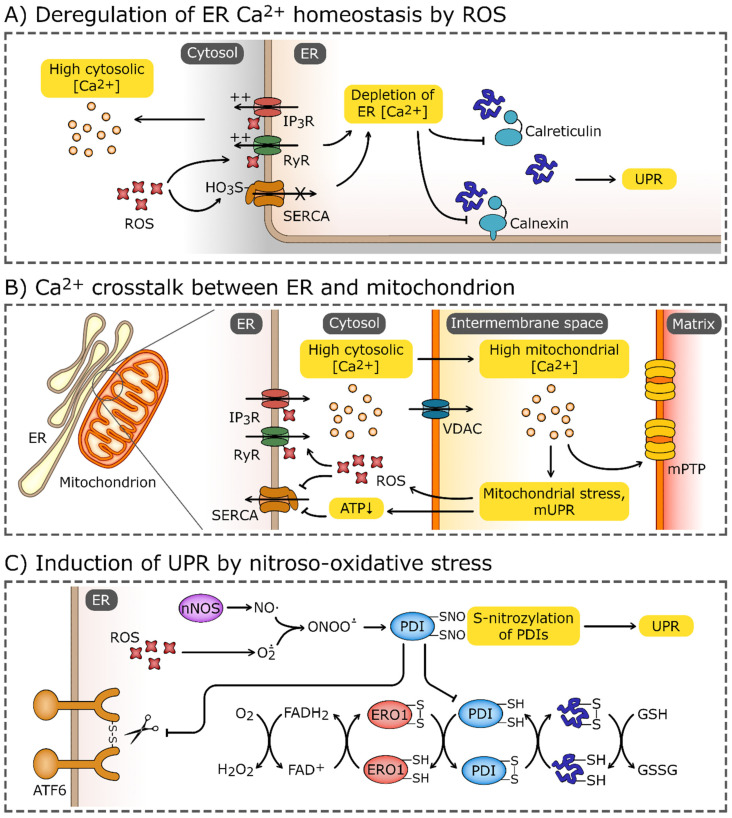
Induction of UPR by oxidative stress. (**A**) Elevated reactive oxygen species (ROS) levels may cause the oxidation of endoplasmic reticulum (ER) calcium transporters, most notably, ryanodine receptors (RyRs), and inositol-1,4,5-trisphosphate (IP_3_) receptors (IP_3_Rs). Elevated ROS levels also promote sulfoxidation of Cys674 of sarco/endoplasmic reticulum Ca^2+^ ATPase (SERCA). These modifications lead to efflux of Ca^2+^ from ER and impairment of Ca^2+^-dependent chaperons, calnexin and calreticulin. (**B**) The disturbance of ER Ca^2+^ homeostasis may spread through mitochondria-associated membranes and target the mitochondrion, causing the Ca^2+^ influx through the voltage-dependent anion channel (VDAC). High Ca^2+^ concentrations induce mitochondrial stress, which leads to activation of the mitochondrial unfolded protein response (mUPR) and formation of mitochondrial permeability transition pores (mPTP). Increased leakage of ROS from electron transport chains and depletion of ATP enhances further ER stress and deregulation of Ca^2+^ homeostasis. (**C**) Increased ROS concentrations combined with the production of NO by nNOS (neuronal nitric oxide synthase) leads to the formation of peroxynitrate (ONOO^-^) which reacts with thiol group of proteins. S-nitrosylation inhibits the activity of modified proteins, including protein disulfide isomerases (PDIs). PDIs, accompanied by ER oxidoreductin 1 (ERO1), catalyze the formation and cleavage of disulfide bonds, and are one of the crucial components of the ER proteostasis system. The reduced-to-oxidized ratio of glutathione (GSH/GSSG), which plays a role analogous to PDIs, may also be increased by the oxidative environment in ER. PDIs also directly affect the UPR sensors and activate transcription factor 6 (ATF6) and inositol-requiring protein 1α (IRE1α).

Increased cellular oxidation can disrupt ER homeostasis and trigger UPR activation and eventually lead to cell death. These oxidative insult-related ER stressors include deregulation of ER calcium homeostasis, nitrosative stress, and mitochondrially generated ROS, as well as ischemic events, discussed below [191,192,193,194,195,196]. Calcium homeostasis is a critical component here (Figure 3). Calcium influx to the ER is mediated by pumps from the sarco/endoplasmic reticulum calcium transport ATPase (SERCA) family, whereas the efflux occurs via the inositol 1,4,5-trisphosphate (IP_3_) receptors (IP_3_R) channels, the ryanodine receptor (RyR) channels, and a heterogeneous collection of calcium leak pores [28,197,198]. Importantly, although sulfoxidation of cysteine 674 in SERCA will prevent calcium influx to ER, the nitric oxide-mediated glutathionylation of this cysteine residue has an opposite effect [191,199,200]. These independent reports stress the importance of maintaining proper redox homeostasis in terms of ER calcium storage. Furthermore, ROS-dependent posttranslational modifications of IP_3_R and RyR channels enhance calcium efflux from ER and consequently impair the calcium-dependent protein folding machinery (calnexin and calreticulin) and lead to the activation of UPR [89,201,202].

Calcium depletion of ER can also be attributed to the crosstalk between the ER and mitochondria and the fact that efficient calcium influx to the ER requires ATP. Hence, oxidative stress-related alterations of the mitochondrial calcium pool and function may impair ER calcium balance and activate the UPR (Figure 3B). Mitochondrial associated membrane (MAM) regions of the ER are known to amplify calcium release and signaling [36,203]. Furthermore, the increased release of mitochondrial H_2_O_2_ also stimulates ER calcium release via the oxidation of IP_3_ receptors [201]. Disturbed MAM signaling has been associated with both Alzheimer’s disease (AD) and amyotrophic lateral sclerosis (ALS), neurodegenerative diseases that are associated with ER stress [204,205]. Additionally, IP_3_R channels are regulated by the ER membrane presenilins that are also considered ER calcium leak channels [206,207], and mutations in the presenilins are associated with AD [208,209,210,211]. Although the role of presenilins in maintaining ER calcium homeostasis requires further study, some of the mutations in these proteins were shown to disturb UPR signaling [212].

In neurons, oxidative stress-related damage results in reduced ATP and NADH synthesis and eventually impairment of complex I that leads to increased levels of O_2_^·-^ [213]. This leads to ER stress and activation of the apoptotic branch of the UPR, including the ER-stress associated caspase 12 [193,214,215,216]. Furthermore, the increase in mitochondrial ROS (both O_2_^·-^ and H_2_O_2_) along with the NO synthesized by nNOS can result in formation of peroxynitrite (ONOO^-^) [217] and leads to the formation of S-nitrosylated proteins [218]. Notably, PDIs that facilitate proper disulfide bond formation and rearrangements in ER can be S-nitrosylated, and if so, their activity is inhibited and leads to the accumulation of misfolded polyubiquitinated proteins in ER and activation of the UPR [219,220]. Since increases in PDI activity serve as a neuroprotective mechanism preventing accumulation of immature and misfolded proteins upon ischemia and during neurodegenerative disorders, the oxidative stress-related impairment of these ER resident chaperones can dramatically influence neurodegeneration [221].

Ischemic events in the brain affect mitochondrial function and result in elevated ROS levels and limit ATP production. This would therefore inhibit energy-dependent cellular functions including the maintenance of ion homeostasis and the redox potential [222,223,224,225,226,227]. Notably, the ischemic ATP level reduction is accompanied by the accumulation of NADH and acyl esters of coenzyme A and carnitine, and these acyl esters were shown to impair both mitochondrial function and structure [228,229]. These changes would impair protein and lipid synthesis, as well as protein folding in ER, and therefore activate the UPR and UPRmt [88,166,167,168,169]. An unmet oxygen cellular demand results in increased levels of BiP as well as PERK activation [95,230,231,232,233,234,235,236,237,238,239,240,241,242]. This suggests that reduced ATP production due to hypoxia or mitochondrial dysfunction can be at least partially counteracted by reducing global translation by an integrated stress response, whereas the related ATF4 signaling restores the mitochondrial and ER balance [166,167,168,169,177].

Although mild and short-lived ischemic events are well controlled by hypoxia-inducible factors (HIFs) that allow both adaptation and survival of neural cells and prevent extensive ROS formation [243,244,245,246,247,248], the rapid reestablishment of normal oxygen levels is often accompanied by overproduction of ROS and cellular damage that is referred to as ischemia–reperfusion injury [243,244,249,250,251,252,253,254]. This damage is accompanied by hyperoxidation of NADH in some neurons and consequently enhanced generation of O_2_^·-^ and acute oxidative stress [255,256,257]. Not surprisingly, ischemia–reperfusion injury has been also associated with the rapid depletion of ER calcium and extensive activation of UPR and UPRmt [258,259,260,261,262,263,264,265,266,267,268,269,270,271,272,273,274,275,276,277,278,279,280,281,282].

ROS may also react and change properties of other ER-important molecules such as lipids, proteins and nucleic acids and thus impair ER function. For example, mRNA oxidation that has been observed in neurodegenerative diseases, including AD and ALS [61,62,63], can result in ribosome stalking and disturbances of cotranslational folding that could eventually contribute to ER stress [59]. Furthermore, ROS-related lipid oxidation can alter ER membrane composition that may also activate the UPR via IRE1 or PERK [283,284,285,286]. Furthermore, since cholesterol autoxidation is proportional to ROS levels, the oxidative stress can result in increased generation of non-enzymatically produced oxysterol [287] that can also disrupt ER membranes and lead to activation of the UPR [288,289,290].

## 6. ER Stress Contributions to Oxidative Stress

Disulfide bond generation in the ER is an oxidative process that utilizes O_2_ and H_2_O_2_ as the electron acceptors [291,292]. Oxygen is required by oxidases such as ER oxidoreductin 1 (ERO1) [293], whereas H_2_O_2_ is generated by the glutathione peroxidases 7 or 8 (GPX7, GPX8) and peroxiredoxin IV (PRDX4) [292,294,295,296]. These two types of enzymes are involved in disulfide bond generation complement and control each other since ERO1 catalysis results in H_2_O_2_ formation that has to be reduced by GPX7 and GPX8 [297]. Notably, PRDX4 reactions rely on other sources of H_2_O_2_ in ER [297]. PDIs mediate oxidation of cysteine residues in the proteins that require oxidative folding in ER [294]. Although, this oxidative protein folding system is well maintained during normal physiological conditions, during prolonged stress, disulfide bond formation in ER may contribute to oxidative stress through the PERK branch of the UPR [298,299,300]. During chronic stress, the PERK signals switch from the integrated stress response to the propagation of proapoptotic CHOP signaling. The increased expression of some CHOP target genes such as *ERO1* may contribute to enhanced ROS generation in ER. Upon ER stress, the expression of GPX8 peroxidase increases as well [297], and thus the importance of CHOP-ERO1 axis in inducing oxidative stress in vivo remains unclear. Other studies, however, have indicated that increased ERO1 levels can result in increased efflux of ER calcium through IP_3_R channels [301,302], and these in turn activate the JNK pathway and stimulate ROS production by the oxidases NOX2 and NOX4 [303,304]. Consequently, ERO1-mediated efflux of ER calcium leads to oxidative stress and amplifies CHOP signaling [303,304]. Furthermore, the ER-stress related increase in H_2_O_2_ generation leads to elevated oxidized GSH levels and thus further reduces the cellular ROS buffering capacities [299,305].

More importantly, chronic or exaggerated ER stress results in dramatic ER calcium efflux as well as activation of UPR apoptotic signaling that can support mitochondrial ROS release and lead to oxidative stress [146]. As mentioned, UPR-induced intrinsic apoptosis relies on B-cell lymphoma 2 (BCL2) repression and induction of BH3-only proteins, including the BCL-2 interacting mediator of cell death (BIM), NOXA, PUMA, death receptor 5 (DR5), and proto-oncogene c (CRK) [82,306,307,308,309,310,311]. Such a programmed increase in mitochondrial outer membrane permeability allows the release of cytochrome c, changing the gating of mPTPs, and the balance between ER and mitochondrial calcium pools, all of which leads to mitochondrial dysfunction and ROS generation [207,312,313]. Furthermore, ER stress-related increases in cytosolic calcium may stimulate phospholipase A_2_ activity and consequently enhance peroxidation of unsaturated lipids and contribute to oxidative stress [314,315].

**Figure 4:**
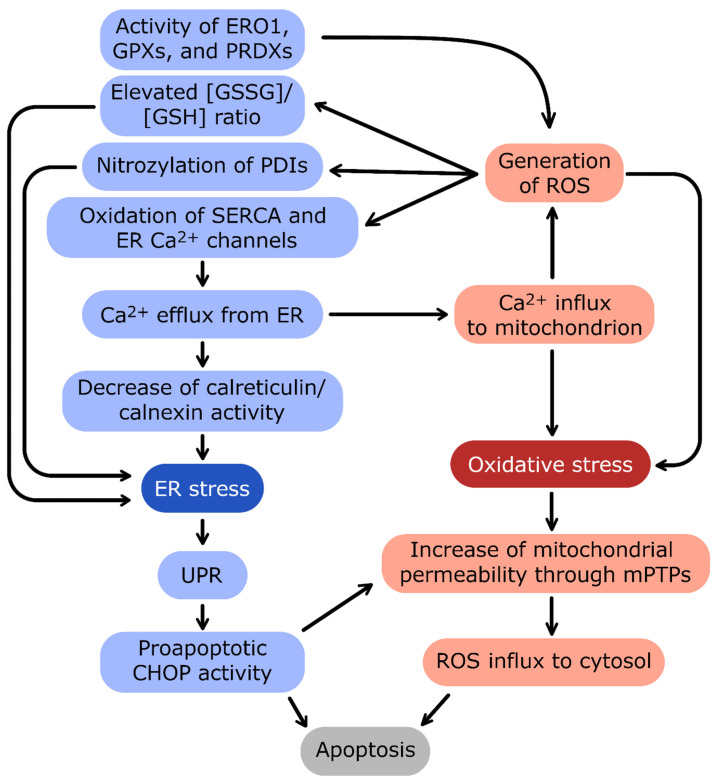
The crosstalk between ER stress and oxidative stress. The main linkage between endoplasmic reticulum (ER) and mitochondrion homeostasis is the Ca^2+^ concentration interdependence.

Taken together, depending on the pathological situation, the chronic or exacerbated activity of this pathway caused by accumulation of mutated misfolded proteins in neurodegenerative diseases such as AD and ALS can also induce ROS production (Figure 4) [64,316].

Ca^2+^ efflux from ER and influx into mitochondria are connected by a positive-feedback loop: oxidative stress and reactive oxygen species (ROS) generation induce the release of Ca^2+^ from ER, and in turn, high Ca^2+^ stimulates the oxidative stress. The important source of ROS in ER is the activity of enzymes catalyzing redox reactions: protein disulfide isomerases (PDIs), ER oxidoreductin 1 (ERO1), glutathione peroxidases (GPXs), and peroxiredoxins (PRDXs). ER stress induces activation of proadaptive unfolded protein response (UPR). In the case of prolonged and excessive stress, UPR activates apoptotic transcription factor CCAAT/enhancer-binding protein homologous protein (CHOP) and severe oxidative stress leads to formation of mitochondrial permeability transition pores (mPTPs). Both pathways trigger eventual apoptosis of cells during unmitigated cellular stress conditions.

## 7. Discussion

Given the complexity of the processes described above and challenges that the brain cells experience while maintaining oxygen homeostasis, it is important to understand molecular mechanisms that assure their proper functioning and survival, as well as the role that the ER plays in this regulation.

Although it seems obvious that oxidative stress accompanies brain pathologies and aging, the role of proadaptive stage of UPR pathway remains underappreciated both in research and clinical approaches. The majority of current approaches focus on the elimination of death-related signals during chronic ER stress, and that is understandable given the pathomechanisms of many of the neurodegenerative diseases, including ALS, AD, Parkinson’s disease (PD), and prion diseases [64,316,317,318,319,320,321,322,323,324]. In these cases, the chronic ER stress will have devastating effects on cell survival. Notably, some studies have shown the benefits of supporting adaptive UPR activity in these disease models. For example, the neuroprotective effects of the transgenic increased levels of XBP1s in a PD mice model [325] and the use of chemical chaperones such as 4-phenyl butyric acid (4-*PBA*) to reduce stress [326]. Furthermore, the forced activation of ATF6 in forebrain neurons improved functional recovery in a mouse model of stroke and Huntington’s disease [327,328].

Alternatively, UPR-inhibiting approaches have also been tested. The PERK pathway inhibitor ISRIB [329] was able to attenuate amyloid β-induced neuronal cell death in AD [330], and was also shown to be promising for therapies targeting ALS [331] and traumatic brain injury (TBI) [332]. Furthermore, the “free radical theory of aging” proposes that the long-term accumulation of oxidative stress incidents will eventually manifest itself by impairing the cellular abilities of maintaining homeostasis, including mitochondrial and ER function [333,334]. Although ROS scavengers seem like a straightforward strategy to cope with neurodegeneration, successful approaches to improve aging-related declines in cognitive function in humans with antioxidants are rarely successful [335,336]. Furthermore, similar limitations were observed during clinical trials using antioxidant strategies in stroke or cardiac ischemia [28,198,337,338,339,340,341]. The main challenges of antioxidant therapies are related to the short half-life of ROS, and this requires scavenger molecules to be extremely efficient, lipid-permeable, and usually used at very high concentrations [28,342].

Thus, development of effective strategies against neurodegeneration and aging requires extension of therapeutic strategies towards other mechanisms that regulate brain cell homeostasis, including the UPR. Notably, a recent study showed the importance of proper balance between the proadaptive and proapoptotic activity of IRE1 in aging brain by demonstrating that XBP1 expression alleviated many of the age-related functional changes [343]. Furthermore, activation of PERK signaling may also have neuroprotective effects [344].

## 8. Conclusions

Here, we have discussed how regulation of the UPR in the ER and mitochondria deals with oxidative stress, how the two collaborate to regulate redox homeostasis, and how things can go wrong with the high oxygen demands of neuronal cells. More insight, however, is needed to understand how these pathways can be manipulated to control the key translations between the survival and death pathways. Both sides of the UPR pathways need to be considered. The findings discussed here emphasize the role of the adaptive ER stress responses for preserving proper brain cell homeostasis. This suggests that reprograming the UPR pathways in order to increase the cellular survival pathways rather than the apoptotic pathways should be tested. Only a precise understanding of mechanisms governing both brain cell redox homeostasis and its crosstalk with UPR_mt_ and the ER UPR will lead to effective therapies for age-related cognitive decay and neurodegenerative diseases.
